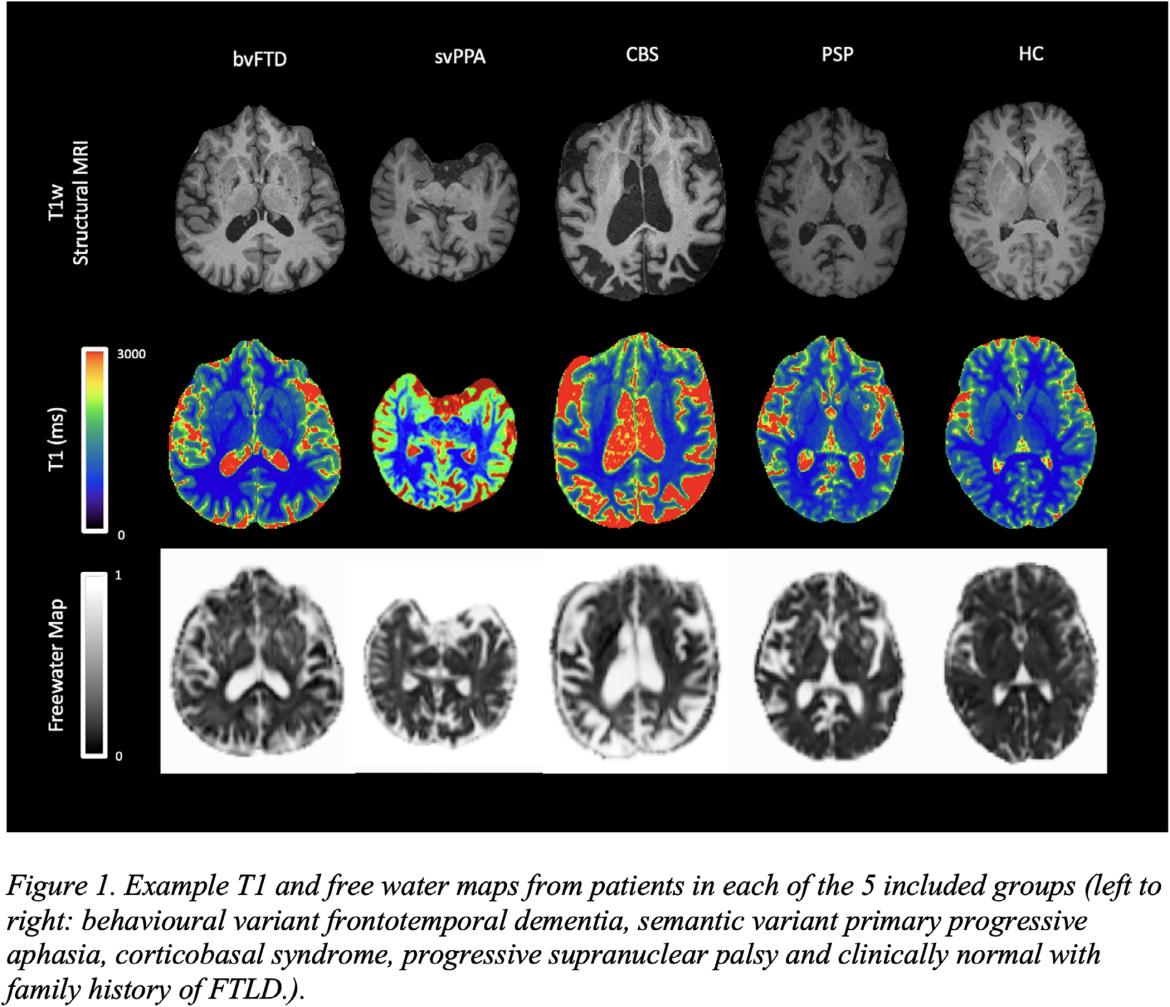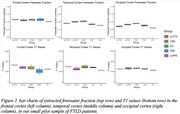# Free‐water Diffusion and Quantitative T1 mapping in FTLD

**DOI:** 10.1002/alz70862_109802

**Published:** 2025-12-23

**Authors:** Vishaal Sumra, Sriranga Kashyap, Kamil Uludag, Kamil Uludag, Nico Paulo Dimal, Alonso Morales‐Rivero, Abeer Khoja, Carmela Tartaglia

**Affiliations:** ^1^ University of Toronto/ University Health Network, Toronto, ON Canada; ^2^ Krembil Brain Institute, Toronto Western Hospital, University Health Network, Toronto, ON Canada; ^3^ Krembil Brain Institute, Sunnybrook Research Institute, Toronto, ON Canada; ^4^ Memory Clinic, Toronto Western Hospital, University Health Network, Toronto, ON Canada; ^5^ University Health Network, Toronto, ON Canada; ^6^ Toronto Western Hospital, University Health Network Memory Clinic, Toronto, ON Canada; ^7^ The Edmond J. Safra Program in Parkinson’s Disease and Morton and Gloria Shulman Movement Disorders Clinic, Toronto, ON Canada; ^8^ Toronto Western Hospital, Tanz Centre for Research in Neurodegenerative Disease, Toronto, ON Canada; ^9^ Division of Neurology, Toronto Western Hospital, University Health Network, Toronto, ON Canada

## Abstract

**Background:**

Neuroinflammation has been proposed as a common feature of neurodegenerative diseases (NDs). Differences in inflammatory profiles have been observed between NDs suggesting disease specific inflammatory profiles. In frontotemporal lobar degeneration (FTLD), neuroinflammation and proteinopathy are expected in frontal and temporal regions, depending on subtype. Free‐water diffusion (FWD) and T1 maps are non‐specific biomarkers of neuroinflammation, where increased tissue water caused by cytokine release is expected to increase both FWD and T1 values. Here we investigate T1 mapping and FWD as candidate neuroinflammatory biomarker in FTLD.

**Method:**

Data was acquired in 25 subjects: 8 corticobasal syndrome (CBS, 4M 4F, mean age 65), 3 semantic variant primary progressive aphasia (svPPA, 3F, mean age 80), 4 progressive supranuclear palsy (PSP, 3F, 1M mean age 77), 7 behavioural variant frontotemporal dementia (bvFTD) (6M,1F mean age 68), and 3 healthy control subjects with a family history of FTLD but gene negative (1M,2F, mean age 52). T1‐weighted MPRAGE, diffusion‐weighted EPI (two shells) for free‐water mapping, and MP2RAGE for qT1 mapping were acquired on a Siemens MAGNETOM Prisma 3T scanner. Skull stripping and binary mask creation were done using ICVmapp3r on T1‐weighted MPRAGE scans. MP2RAGE T1 maps were corrected for B1+ using a separately acquired B1+ map. FWD maps were processed with Synb0, FSL's topup, FSL's eddy, and in‐house MATLAB to generate final FWD maps. FWD and T1 maps were co‐registered using FSL flirt. FWD and T1 values were extracted in the caudate, as well as frontal and temporal regions where FTLD pathology is expected.

**Result:**

FWD maps show good contrast between tissue and CSF, whereas T1 maps show good contrast between grey matter, white matter and CSF. We observe trends in the expected directions with increased T1 and FW in frontal vs occipital regions in PSP, bvFTD, CBS and svPPA patients, and increased FW and T1 in the frontal cortex in bvFTD, CBS, and PSP patients compared to clinically normal individuals.

**Conclusion:**

Our preliminary results suggest that both FWD and qT1 are increased in areas where pathology is expected in FTLD syndromes. Further studies will as examine the relationship between FWD, qT1 and biofluid based neuroinflammatory biomarkers.